# Association of a polymorphism in PON-1 gene with steroid-induced osteonecrosis of femoral head in Chinese Han population

**DOI:** 10.1186/1746-1596-8-186

**Published:** 2013-11-08

**Authors:** Zhiyao Wang, Yanqiong Zhang, Xiangying Kong, Shangzhu Li, Yimin Hu, Rongtian Wang, Yan Li, Chao Lu, Na Lin, Weiheng Chen

**Affiliations:** 1 Wangjing hospital (Hospital of Orthopedics and Traumatology), China Academy of Chinese Medical Sciences, Beijing 100102, China; 2 Institute of Chinese Materia Medica, China Academy of Chinese Medical Sciences, Beijing 100700, China; 3 Institute of Hematology & Blood Diseases Hospital, Chinese Academy of Medical Sciences & Peking Union Medical College, Tianjin 300041, China

**Keywords:** Steroid-induced osteonecrosis of the femoral head, Paraoxonase-1, Single nucleotide polymorphism

## Abstract

**Background:**

Treatment with steroids covers a wide spectrum of diseases in clinic. However, some users are suffering from serious side effects of steroid administration, while we enjoy the benefit it brings about. Osteonecrosis of the femoral head (ONFH) is a troublesome one among them. Recent studies have demonstrated that lipid metabolism disorder may play a vital role in pathogenesis of ONFH and mutation of the paraoxonase-1 (PON-1) gene may be involved in the occurrence of this disease. However, the relationship between polymorphisms of PON-1 and ONFH has not been thoroughly studied. The aim of this study was to determine whether PON-1 polymorphisms are associated with steroid-induced ONFH through a cohort study among Chinese Han population.

**Methods:**

This trial applied a case–control scheme to compare the clinical data including PON-1 SNP among 94 patients and 106 control subjects to analyze the association between SNP and risk of steroid-induced ONFH. Time of Flight Mass Spectrometer is utilized for genotyping and the result was analyzed in multivariate analysis models.

**Results:**

According to polymorphism test of rs662, its SNP was significantly associated with the risk of ONFH in overdominant analysis model [P value: 0.022; odds ratio (OR): 0.39]. However, genotype frequencies of rs662 of PON-1 gene between case and control group showed no differences (P > 0.05).

**Conclusions:**

Our data suggest for the first time that SNP (rs662) of the PON-1 gene was associated with the risk of steroid-induced ONFH. In addition, PAI-1 SNPs may play an important role in pathogenesis of ONFH.

**Virtual slides:**

The virtual slide(s) for this article can be found here: http://www.diagnosticphatology.diagnomx.eu/vs/1501829501107336.

## Introduction

Osteonecrosis of femoral head (ONFH) is a bone disease that cellular death happens within femoral head owing to damage of blood supply to the anterior–superior– lateral part of the femoral head [1]. Without effective intervention, most cases will develop collapse of femoral head and eventually degenerative arthritis of the hip [2]. Although quite a number of treatments were developed to relief or reverse the course of this illness, none of them is satisfactory to solve this intractable medical condition. Invention of total hip arthroplasty is a milestone in management of ONFH, however, the patients often require multiple increasingly difficult surgeries over the course of a lifetime because the average age at presentation is very young (about 33 years of age).

Lots of factors as trauma, alcoholism, coagulation defects, hematopathy, certain autoimmune diseases may lead to osteonecrosis. Beyond that, some treating modalities also increase the risk of this disease. For example, Bisphosphonate contributes to osteonecrosis of jaw (BRONJ) as well as steroids may induce osteonecrosis in multiple joints including hip joint [3]. Among ONFH caused by different etiologies, steroid-induced ONFH attracts vast attention for following reasons: first, there is a growing trend of its morbidity and some epidemic studies suggests it has became the leading cause of ONFH; Second, it typically happens in young patients that is the worst cases to handle; Last, preventative methods seems effective due to laboratory trial [4]. From the fact that even receive same steroid administration regime, not every patient develop ONFH, it is easy to reach the conclusion that steroid sensitivity are different among people. Hence, it is imperative for medical community to bring about substantial moves in prevention. Early perception of susceptible signs in patients on steroid medication is needed for its prospective benefits. To address this problem, the etiology and pathogenesis of ONFH must be thoroughly surveyed to find a starting point. According to current condition, it is neither practicable nor necessary to apply preventative modality on the all patients medicated by steroid, thus screening for susceptible population seems to be the key point making prevention more efficient. With the progress of molecular biology, increasing number of gene was found to be associated with certain disease. Further studies combined several functional genes into a model to predict the occurrence of cancer which make it even closer to clinical application [5]. Presently, single nucleotide polymorphism (SNP) arouses vast interests and is believed to be the most promising direction in this field. In addition to sound proof favoring SNP is related to disease-susceptibility, it also possesses a merit called dimorphism which means each locus comprises of two alleles—it is either this or that. That leads to low cost and difficulty of detect and screen which helps SNP to become a hot spot in medical research. Since its advantages mentioned above, SNP is widely used in research involving steroid-induced ONFH.

To our knowledge, the specific mechanism of steroid-induced ONFH is still unclear. Several hypotheses concerning fat embolism, intraosseous hypertension and venostasis, microvascular impair and osteoporosis are proposed according to pathological observation and they are deemed as reliable ones [6-9]. Apparently lipid metabolism abnormality as well as coagulation disorder are two crucial pathogenic states facilitating this disease. Regarding lipid metabolism abnormality, it refers to elevation of LDL, decrease of HDL and etc. Amount of researches have demonstrated that hyperlipidimia commonly happened in steroid-induced ONFH both in laboratory and clinic [10]. Lipid metabolism is a complicated series of reactions affected by many substances and paraoxonase (PON) is one of them which deeply involved its course. The genetic polymorphism of this enzyme was found to be linked to hyperlipidemia [5]. Actually, the paraoxonase (PON) multi-gene family consists of at least three members as PON-1, PON2 and PON3 that map to 7q21.3-22 of chromosome7 [11]. All of the three members participate the proatherogenic oxidative modification of low-density lipoprotein (LDL) and cell membranes and are therefore considered to be anti-atherogenic while only PON-1 is believed to be the major factor in the antioxidative activity of HDL [12,13]. Plymorphyism of the PON-1 gene causes variation of blood levels of PON-1 and its catalytic efficacy leading to susceptibility to therosclerosis [14]. PON-1 has two common polymorphisms at codon 192 [A/G: Gln (Q)/Arg (R)] and 55 [T/A: Leu(L)/Met (M)]. It has been proven that the variation and enzyme activity has a cause and effect relationship [15]. In certain populations, elevated enzyme activity gives birth to higher HDL level [16]. Recent researches mainly focus on PON 192 and 55 polymorphisms, several trials found that they were associated with risk of ischemic vascular events as stroke and coronary heart disease [17]. One study also showed that PON-1 192QQ genotype increased risk for steroid-induced ONFH [18]. On the basis of these findings, we hypothesized that there might be a possible relationship between SNP of PON-1 and steroid-induced ONFH. In order to validate this hypothesis, we performed the current study by a cohort study among Chinese Han population.

## Materials and methods

### Subjects

This study was approved by the ethic committee of two hospital involved—namely Wangjing Hospital, China Academy of Chinese Medical Sciences and Hematology & Blood Diseases Hospital,Chinese Academy of Medical Sciences. And informed consent of all individuals enrolled in the study were obtained after they were full informed what they were engaging in.

A total of 94 patients with steroid-induced ONFH (case group, 40 men, 54 women; mean age: 40.22 ± 14.78 years) and 106 patients who did not develop steroid-induced ONFH (reference group, 64 men, 42 women; mean age: 43.09 ± 18.36 years) following steroid administration were consecutively enrolled at Wangjing Hospital and Institute of Hematology & Blood Diseases Hospital of China Academy of Chinese Medical Sciences from March 2011 to December 2012. All the subjects are from 14 provinces in China, including Hebei (n = 41), Beijing (n = 32), Tianjin (n = 27), Shandong (n = 21), Henan (n = 18), Liaoning (n = 14), Jilin (n = 11), Shanxi (n = 10), Heilongjiang (n = 10), Neimenggu (n = 8), Guizhou (n = 3), Ningxia (n = 2), Shanxi (n = 2) and Qinghai (n = 1). Steroid-induced ONFH was defined by a history of a mean daily dose of ≥ 16.6 mg or highest daily dose of 80 mg of predinosolone equivalent within 1 year prior to the development of symptoms or radiological diagnosis in asymptomatic cases (Oinuma et al. 2001; Koo et al. 2002; Mont et al. 2006) [19-21]. Underlying diseases in steroid-induced ONFH were hematologic diseases (n = 23 patients), dermatogic diseases (n = 9 patients), renal diseases (n = 9 patients), ophthalmopathy (n = 6 patients), diseases of respiratory system (n = 5 patients), and others (n = 42 patients). Patients with a demonstrable history of direct trauma or with possible combined causes were excluded. The clinical characteristics of patients in case and reference groups were summarized in Table 1.

**Table 1:** Clinical information

**Factor**		**Steroid-induced ONFH Cases (N = 94)**	**Non steroid-induced ONFH Cases (N = 106)**	**P**
Gender	Male	40	64	0.02
	Female	54	42	
Age		40.22 ± 14.78	43.09 ± 18.36	0.23
Body mass index (BMI, Kg/m2)		23.01 ± 3.00	24.36 ± 2.42	< 0.001
Affected sides	Unilateral	15	--	
	Bilateral	79	--	
Steroid treatment	Oral medication	74	89	0.06
	Intravenous injection	36	71	
Steroid dose	Large (> 400 mg/d)	34	60	0.12
	Middle (> 100 mg/d & < 400 mg/d)	52	40	
	Small (< 100 mg/d	8	6	
Treatment duration (days)		1140.34 ± 2014.037	2631.54 ± 8610.278	0.10
Underlying diseases (reason for steroid)	Hematologic diseases	23	106	< 0.001
	Dermatogic diseases	9	0	
	Renal diseases	9	0	
	Ophthalmopathy	6	0	
	Respiratory disease	5	0	
	others	42	0	

### Candidate PON-1 single nucleotide polymorphism (SNP) selection

A well-studied functional SNPs in the PON-1 gene, rs662 (−844 G/A, in the promoter) was selected in the list of genotyping in this study for its important functions in regulating PON-1 expression and in the development of ONFH. As the results of our literature retrieval from PubMed database (http://www.ncbi.nlm.nih.gov/pubmed), a study showed for the first time that rs662 was essential SNP involved in the regulation of PON-1 gene expression in ONFH using a Greek population [18]. According to their analysis, they confirmed the risk effects of rs662 in the non-traumatic ONFH. In the current study, the relationship between rs662 SNP and the development of steroid-induced ONFH were investigated using a large cohort of Chinese population.

### DNA isolation

Genomic DNA was extracted from 2 mL whole-blood samples using the QIAamp DNA Blood Mini kit (Qiagen, Inc., Valencia, CA) following the manufacturer’s protocol. After dilution to 20 g/μL, DNA was distributed in 96-well plates and stored at −80.

### PON-1 SNP genotyping

SNP genotyping was performed on the SEQUENOM MassARRAY® Analyzer 4 (Sequenom, Inc., San Diego, CA, USA) using genomic DNA in a single multiplex reaction. Primers for polymerase chain reaction (PCR) amplification and single base extension were designed by Sequenom Assay Design 3.1 software (Sequenom, San Diego, CA, USA) according to the manufacturer’s instructions (Table 2). For quality control, genotyping was performed without knowledge of the case/control status of the subjects, and a random sample of 5% of cases and controls was genotyped again by different researchers. The reproducibility was 100%. The genotyping success rate was over 95%. In this section, researchers were blind to group information of samples they deal with to avoid selection bias.

**Table 2:** Polymerase chain reaction primers of SNP

**SNP**	**PCR primer**	**Sequence**	**Extension primer**	**Sequence**
Rs662	1^st^-Primer	ACGTTGGATGGATCACTATTTTCTTGACCC	1^st^-Primer	TGTTCTTGACCCCTACTTACA
	2^nd^-primer	ACGTTGGATGTAGACAACATACGACCACGC	2^nd^-primer	TGTTCTTGACCCCTACTTACG

### Statistical analysis

Whether individual variants were in Hardy–Weinberg equilibrium was determined by Chi-square tests. Chi-square tests were adopted to assess differences in genotype and allele frequency between case group and control group. Beyond that, statistical significance was judged by the p values obtained from the logistical regression analysis, controlling for age, gender, body mass index (BMI), concurrent disease, steroid dose and duration of steroid administration as covariates with four alternative models (codominant, dominant, recessive and overdominant). All analyses were two-tailed and a p value less than 0.05 were considered to be statistically significant. Statistical analyses were performed by using SAS 9.1 (SAS Institute Inc. Cary, NC, USA) as well as SPSS 12.0 (SPSS Inc, IL, USA).

## Results

### Patient characteristics

Analysis showed that age, steroid treatment, steroid dose, and duration of steroid treatment between case group and control group was similar (all P > 0.05). However, two groups were obviously different from each other in gender, (P = 0.02), BMI (P < 0.001) and concurrent disease (P < 0.01). More Male patient suffered from ONFH among these steroid users while more patients with relative less BMI developed ONFH.

### Polymorphism of rs662 was associated with the risk of steroid-induced ONFH

The genotype frequency of rs662 polymorphism was in accordance with Hardy-Weinberg equilibrium in both case and control groups (Table 3). The P values of polymorphism were analyzed by logistic analysis with respect to a comparison between steroid-induced patients and the controls.

**Table 3:** Exact test of rs662 for Hardy-Weinberg equilibrium (n = 195)

	**N11**	**N12**	**N22**	**N1**	**N2**	**P-value**
**All subjects**	68	97	30	233	157	0.77
**co.ca = 0**	30	57	15	117	87	0.22
**co.ca = 1**	38	40	15	116	70	0.51

As shown in Table 4, the rs662 allele and genotype frequencies showed a significantly higher risk factor in ONFH patients compared with those in controls with P values 0.022 (OR; 0.39, 95% CI; 0.17-0.89) in the overdominant model. Though the P value of rs662 genotype frequency in the condominant model was slightly greater than 0.05 (P = 0.072), it showed the tendency of weak association with the risk of ONFH development. No differences in the frequencies of the allele and genotypes were seen in patients and the controls in the case rs662 polymorphisms.

**Table 4:** Association between rs662 SNPs and the risk of steroid-induced ONFH

**Model**	**Genotype**	**co.ca = 0**	**co.ca = 1**	**OR (95% CI)**	**P-value**	**AIC**	**BIC**
Codominant	G/G	23 (27.1%)	27 (38.6%)	1.00	0.072	189.5	235.1
	A/G	49 (57.6%)	30 (42.9%)	0.38 (0.14-1.01)			
	A/A	13 (15.3%)	13 (18.6%)	0.95 (0.29-3.14)			
Dominant	G/G	23 (27.1%)	27 (38.6%)	1.00	0.13	190.4	233
	A/G-A/A	62 (72.9%)	43 (61.4%)	0.49 (0.20-1.24)			
Recessive	G/G-A/G	72 (84.7%)	57 (81.4%)	1.00	0.23	191.3	233.9
	A/A	13 (15.3%)	13 (18.6%)	1.83 (0.67-5.02)			
Overdominant	G/G-A/A	36 (42.4%)	40 (57.1%)	1.00	*0.022*	187.5	230.1
	A/G	49 (57.6%)	30 (42.9%)	0.39 (0.17-0.89)			
Log-additive	---	---	---	0.92 (0.51-1.66)	0.78	192.7	235.3

## Discussion

Although collapse of femoral head is the distinctive feature in radiography which is easy to be identified, it commonly happens in the middle stage. Before that, bone marrow edema, cystic degeneration, bone density loss and other manifestations are all nonspecific signs. Then, the forthcoming subchondral fracture (crescent signs) and calcification zone is reasonable proof for diagnosis but they also mean that the best time for treatment has been past. In addition, pain-the major symptom in early stage, may occurs in lower back, hip or even knees that makes it even more difficult to be diagnosed. Histopathological study reveals that, steroid-induced ONFH suffered diffusive lesions in whole femoral head. Due to the inhibiting effects of steroid on osteoblast the bone repairing course becomes much slower. Furthermore, insufficient blood flow caused by steroid further compromise the repairing process. Consequently, the collapse may develop much faster. Take into account these unfavorable characteristics of steroid-induced ONFH, predicting factors of this disease are needed for prevention and early intervention.

This study was conducted to identify a genetic factor contributing to onset of steroid-induced ONFH. The result indicated that, under the overdominant model, A/G carriers were less susceptible to steroid-induced ONFH than A/A and G/G carriers among patients managed by steroid, suggesting that A/G carriers may have a lower sensitivity to GC than A/A and G/G carriers. In a word, our data suggest for the first time that SNP (rs662) of the PON-1 gene was associated with the risk of steroid-induced ONFH.

As mentioned above, accumulating studies were performed to investigate the relationship between SNP of rs662 (Q192R) and other vascular ischemic diseases. However, their results were not consistent. Two cohort studies in Britain which found no evidence that PON-1 Q192R polymorphism is associated with CHD risk in Caucasian women or men [22,23]. However, the investigation in Thai population turned out to be opposite—a firm association between polymorphism of Q192R and CHD was observed [24]. For stroke, a study in Chinese population showed that Q192R polymorphism (the R allele and RR genotype) was associated with an increased risk of ischemic stroke [17]. On the contrary, a research in Greece indicated that no links between Q192R and stroke was detected [25,26]. The demographic factors as ethic background may contribute to this paradox.

There are several highlights in the current study as following: first, PON-1 polymorphism has been a hot issue, however, very few researches was designed to investigate its relationship with ONFH; Second, differences in PON-1 activity, concentration and genotype distribution have long been known to occur between different populations and geographical regions throughout the world. Accordingly, this is deemed as one of the major controversies between studies concerning relationship between PON-1 polymorphism and other diseases. In this study, all participants were confined as Han Chinese lived in north areas of the Yellow River that is designed to avoid deviation caused by ethical and geographical factors. Last but not the least, unlike some other studies that using healthy people as controls, this research recruit steroid users without developing ONFH as reference group. This scheme is of an obvious advantage that it excludes the influence of steroid—a pathogenic factor on subjects of different groups. Consequently, it increases the reliability of the result.

However, there are several limitations in this study. First, the sample size is relative small, which may influence the reliability of our results because the heterogeneity of concurrent diseases of subjects may lead to selection bias of the result. Second, it is still unknown that by which way it facilitates ONFH. Though PON-1 polymorphism is linked to Atherosclerosis, it could not explain its relationship with steroid-induced ONFH. Not like the chronic course of the former one, the latter one is a medical condition happened much faster. Therefore, the exact mechanisms by which the polymorphisms of the PON-1 gene are involved in the development of steroid-induced ONFH require further investigations. And its result may provoke more deep understanding on safe protocol of steroid administration.

In conclusion, our data provide the convincing evidence for the first time that rs662 SNP of PON-1 may be associated with the risk of steroid-induced ONFH, suggesting that the genetic variations of this gene may play an important role in the disease development. Following study is needed for its prospecting result.

## Competing interests

The authors do not have any conflict of interests with the content of the paper.

## Authors’ contributions

ZW: drafted the manuscript, completed the inclusion and exclusion of case group, YZ: helped drafting the manuscript, interpreted the statistical result, XK: processed the blood samples and extracted DNA, SL: completed the inclusion of control cases, YH: collected information and blood samples of control group, RW: performed the statistics and provided clinical background, YL: collected clinical information of case group, CL: collected involving references, helped drafting the tables, NL, WC: designed the whole study, supervised the course of study, revised the manuscript. All authors read and approved the final manuscript.

## References

[B1] Atsumi T Kuroki Y Role of impairment of blood supply of the femoral head in the pathogenesis of idiopathic osteonecrosis Clin Orthop Relat Res 1992 277 22 30 1372850

[B2] Assouline-Dayan Y Chang C Greenspan A Pathogenesis and natural history of osteonecrosis Semin Arthritis Rheum 2002 32 2 94 124 12430099

[B3] Petcu EB Ivanovski S Wright RG Bisphosphonate-related osteonecrosis of jaw (BRONJ): an anti-angiogenic side-effect? Diagn Pathol 2012 7 78 doi: 10.1186/1746-1596-7-78 10.1186/1746-1596-7-78 22770117 PMC3479027

[B4] Iwakiri K Oda Y Kaneshiro Y Effect of simvastatin on steroid-induced osteonecrosis evidenced by the serum lipid level and hepatic cytochrome P4503A in a rabbit model J Orthop Sci 2008 13 5 463 468 doi: 10.1007/s00776-008-1257-z 10.1007/s00776-008-1257-z 18843462

[B5] Zhang Y Wang S Li D A systems biology-based classifier for hepatocellular carcinoma diagnosis PLoS One 2011 6 7 e22426 doi: 10.1371/journal.pone.0022426 10.1371/journal.pone.0022426 21829460 PMC3145651

[B6] Fisher DE Bickel WH Corticosteroid-induced avascular necrosis: a clinical study of seventy-seven patients J Bone Joint Surg Am 1971 53 5 859 873 4326745

[B7] Wang GJ Dughman SS Reger SI The effect of core decompression on femoral head blood flow in steroid-induced avascular necrosis of the femoral head J Bone Joint Surg Am 1985 67 1 121 124 3968091

[B8] Jones JJ Intravascular coagulation and osteonecrosis Clin Orthop Relat Res 1992 277 41 53 1532547

[B9] Wang GJ Sweet DE Reger SI Fat-cell changes as a mechanism of avascular necrosis of the femoral head in cortisone-treated rabbits J Bone Joint Surg Am 1977 59 6 729 735 908695

[B10] Xiao-yan W Xiao-hong N Wei-heng C Effects of apolipoprotein A1 and B gene polymorphism on avascular necrosis of the femoral head in Chinese population China journal of orthopaedics and traumatology 2008 21 2 99 102 doi: 10.3969/j.issn.1003-0034.2008.02.006 19105467

[B11] Primo-Parmo SL Sorenson RC Teiber J The human serum paraoxonase/arylesterase gene (PON1) is one member of a multigene family Genomics 1996 33 3 498 507 10.1006/geno.1996.0225 8661009

[B12] Mackness B Durrington PN Mackness MI The paraoxonase gene family and coronary heart disease Curr Opin Lipidol 2002 13 4 357 362 10.1097/00041433-200208000-00002 12151850

[B13] Nassar BA Darvesh S Bevin LD Relation between butyrylcholinesterase K variant, paraoxonase 1 (PON1) Q and R and apolipoprotein E epsilon 4 genes in early-onset coronary artery disease Clin Biochem 2002 35 3 205 209 10.1016/S0009-9120(02)00296-5 12074828

[B14] Draganov DI La Du BN Pharmacogenetics of paraoxonases: a brief review Naunyn Schmiedebergs Arch Pharmacol 2004 369 1 78 88 doi:10.1007/s00210-003-0833-1 10.1007/s00210-003-0833-1 14579013

[B15] Humbert R Adler DA Disteche CM The molecular basis of the human serum paraoxonase activity polymorphism Nat Genet 1993 3 1 73 76 doi: 10.1038/ng0193-73 10.1038/ng0193-73 8098250

[B16] Obata T Ito T Yonemura A R192Q paraoxonase gene variant is associated with a change in HDL-cholesterol level during dietary caloric restriction in nondiabetic healthy males J Atheroscler Thromb 2003 10 1 57 62 10.5551/jat.10.57 12621166

[B17] Voetsch B Benke KS Damasceno BP Paraoxonase 192 Gln Arg polymorphism: an independent risk factor for nonfatal arterial ischemic stroke among young adults Stroke 2002 33 6 1459 1464 10.1161/01.STR.0000016928.60995.BD 12052975

[B18] Hadjigeorgiou GM Malizos K Dardiotis E Paraoxonase 1 gene polymorphisms in patients with osteonecrosis of the femoral head with and without cerebral white matter lesions J Orthop Res 2007 25 8 1087 1093 doi: 10.1002/jor.20393 10.1002/jor.20393 17469180

[B19] Oinuma K Harada Y Nawata Y Osteonecrosis in patients with systemic lupus erythematosus develops very early after starting high dose corticosteroid treatment Ann Rheum Dis 2001 60 12 1145 1148 doi:10.1136/ard.60.12.1145 10.1136/ard.60.12.1145 11709458 PMC1753447

[B20] Koo KH Kim R Kim YS Risk period for developing osteonecrosis of the femoral head in patients on steroid treatment Clin Rheumatol 2002 21 4 299 303 doi:10.1007/s100670200078 10.1007/s100670200078 12189457

[B21] Mont MA Jones LC Hungerford DS Nontraumatic osteonecrosis of the femoral head: ten years later J Bone Join t Surg(Am) 2006 88 5 1117 1132 doi: 10.2106/JBJS.E.01041 10.2106/JBJS.E.01041 16651589

[B22] Lawlor DA Day IN Gaunt TR The association of the PON1 Q192R polymorphism with coronary heart disease: findings from the British Women’s Heart and Health cohort study and a meta-analysis BMC Genet 2004 5 17 doi: 10.1186/1471-2156-5-17 15214960 10.1186/1471-2156-5-17 PMC449704

[B23] Wheeler JG Keavney BD Watkins H Four paraoxonase gene polymorphisms in 11212 cases of coronary heart disease and 12786 controls: meta-analysis of 43 studies Lancet 2004 363 9410 689 695 doi: 10.1016/S0140-6736(04)15642-0 10.1016/S0140-6736(04)15642-0 15001326

[B24] Likidlilid A Akrawinthawong K Poldee S Paraoxonase 1 polymorphisms as the risk factor of coronary heart disease in a Thai population Acta Cardiol 2010 65 6 681 691 21302675 10.1080/ac.65.6.2059866

[B25] Liu H Xia P Liu M PON gene polymorphisms and ischaemic stroke: a systematic review and meta analysis Int J Stroke 2013 8 2 111 123 doi: 10.1111/j.1747-4949.2012.00813.x 10.1111/j.1747-4949.2012.00813.x 22631428

[B26] Lazaros L Markoula S Kyritsis A Paraoxonase gene polymorphisms and stroke severity Eur J Neurol 2010 17 5 757 759 doi: 10.1111/j.1468-1331.2009.02860.x 10.1111/j.1468-1331.2009.02860.x 19930448

